# Breastfeeding the late preterm infant: experiences of mothers and perceptions of public health nurses

**DOI:** 10.1186/s13006-017-0114-0

**Published:** 2017-05-08

**Authors:** Aliyah Dosani, Jena Hemraj, Shahirose S. Premji, Genevieve Currie, Sandra M. Reilly, Abhay K. Lodha, Marilyn Young, Marc Hall

**Affiliations:** 1 School of Nursing and Midwifery, Mount Royal University, 4825 Mount Royal Gate SW, Calgary, AB T3E 6K6 Canada; 2 O’Brien Institute of Public Health, University of Calgary, Calgary, AB Canada; 3 Undergraduate Student, University of Calgary, 2500 University Drive NW, Calgary, AB T2N 1N4 Canada; 4 Faculty of Nursing, University of Calgary, 2500 University Drive NW, Calgary, AB T2N 1N4 Canada; 5 Alberta Children’s Hospital Research Institute, Calgary, AB Canada; 6 Department of Paediatrics, Section of Neonatology, Alberta Health Services, Foothills Medical Centre, 1403 29th Street NW, Calgary, AB T2N 2T9 Canada; 7 Prenatal & Postpartum Services, Public Health Calgary Zone, Alberta Health Services, 1430, 10101 Southport Road SW, Calgary, AB T2W 3N2 Canada

**Keywords:** Late preterm infant, Breastfeeding, Mothers’ experiences, Public health nurses’ perceptions, Canada

## Abstract

**Background:**

The promotion and maintenance of breastfeeding with late preterm infants (LPIs) remain under examined topics of study. This dearth of research knowledge, especially for this population at-risk for various health complications, requires scientific investigation. In this study, we explore the experiences of mothers and the perceptions of public health nurses (PHNs) about breastfeeding late preterm infants in Calgary, Alberta, Canada.

**Methods:**

We used an exploratory mixed methods design with a convenience sample of 122 mothers to gather quantitative data about breastfeeding. We collected qualitative data by means of individual face-to-face interviews with 11 mothers and 10 public health nurses. Data were collected from April 2013 to June 2014. We then employed an interpretive thematic analysis to identify central themes and relationships across narratives.

**Results:**

We collected 74 complete data sets about breastfeeding. During the first 6–8 weeks postpartum, 61 mothers breastfed their infants.﻿ Of these, 51 partially breastfed and 10 exclusively breastfed. For qualitative purposes, the researchers interviewed 11 mothers with late preterm babies and three themes emerged: significant difficulty with breastfeeding, failing to recognize the infant’s feeding distress and disorganized behavior, and the parental stress caused by the multiple feeding issues. The public health nurses’ comments reinforced and expanded on what the mothers reported. The themes for the nurses included: challenges with initiating breastfeeding, challenges during breastfeeding, and the need for stimulation during breastfeeding.

**Conclusion:**

Mothers face challenges when breastfeeding their late preterm infants and public health nurses can guide them through this experience. Families with a late preterm infant need to be informed about the challenges associated with breastfeeding a late preterm infant. It is necessary for all health care professionals to receive proper training on safe and effective breastfeeding of late preterm infants. It is essential for public health nurses to communicate effectively with families of late preterm infants to provide anticipatory guidance about potential challenges and strategies to resolve any breastfeeding problems.

## Background

Late preterm infants (≥ 34 ^0/7^ – 36 ^6/7^ weeks gestational age) comprise approximately 75% of all preterm births [1]. Among Canadian provinces, Alberta (2015–2016) has the highest rate of preterm birth (8.6%) and Calgary averages 8.9% [2]. Insofar as late preterm infants (LPIs) face an increased risk for various health complications [3–12], the benefits of breast milk [13–16] become particularly meaningful for this vulnerable population [17]. That is, the bioactive components of breast milk can prove especially crucial for LPIs who “have a compromised immunomodulatory response, have immature organs including the brain, and are susceptible to inflammatory injury and oxidative stress” (p. 690) [18]. However, LPIs have lower exclusive breastfeeding rates than both term infants and early term infants (37–38 weeks gestational age), in part, because families do not receive sufficient and appropriate support in the immediate postpartum period [19].

Briere and colleagues have identified policies and presented recommendations for breastfeeding LPIs that are intended to be used in the acute care setting. However, there is insufficient research evidence currently available that discusses the best approaches to promoting and supporting breastfeeding post discharge [17]. All mothers in Calgary, Alberta, Canada are offered care from public health nurses (PHNs), within 24 to 48 h after discharge, either in the home or clinic setting. Mothers also receive follow-up visit(s) or telephone call(s) as appropriate. Due to the lack of specific breastfeeding protocols for LPIs in the community setting, PHNs typically tailor their experiential knowledge about breastfeeding with term infants or ≥ 34^0/7^ preterm infants when providing anticipatory guidance and practical teaching to mothers of LPIs.

Preterm infants have a decreased likelihood of breastfeeding initiation and shortened breastfeeding duration [20, 21]. To remedy the problem, researchers should address the dearth of research about the specific challenges of breastfeeding LPIs [12, 15, 16]. While the existing literature does present qualitative research about LPIs, our study adds to the literature by exploring mothers’ experiences of breastfeeding LPIs and PHNs’ perceptions about the challenges of providing breastfeeding guidance to mothers. Our research questions were: (1) What is the mother’s experience of caring for LPIs with respect to breastfeeding? And (2) What is the PHN’s experience of caring for LPIs with respect to breastfeeding? This information will help us determine how we may support families during this crucial developmental period.

## Methods

We used an exploratory mixed-methods design, collecting and analyzing both quantitative and qualitative data [22]. Data were collected from April 2013 to June 2014. The process for recruiting mothers for the quantitative component began with PHNs informing mothers of LPIs about the study using a standard script and requested permission to share contact information with the researchers. The researchers approached those mothers who agreed to be contacted and secured informed consent.

We recruited a convenience sample (*n* = 122) of mothers to gather data regarding breastfeeding practices, depression, stress, maternal confidence, anxiety, and social support. This article will focus on breastfeeding practices. Other results of our study can be found elsewhere [23]. Mothers completed a questionnaire about maternal characteristics at delivery, infant characteristics, and demographic information. Missing data were secured, with permission, from the Alberta Health Services administrative database. To promote a high response rate, we personalized cover letters and salutations, provided self-addressed and stamped envelopes, and gave parents an unconditional CAD 20 gift certificate for groceries [24]. Later, we sent a thank you card as a reminder to mail back the package if they had not already done so.

We asked the mothers who participated in the quantitative component for an interview. Of those who agreed, we purposively sampled mothers with varied lengths of hospital stay (e.g., early discharge, prolonged hospitalization), mothers who received services through different models of care (e.g., home visits or individual clinic visits), and families with LPIs at different points of postpartum care (e.g., infant is 1 week, 1 month, and almost 2 months post discharge). When multiple mothers met the criteria, we used random sampling, where names were drawn from a box. If potential study participants were unable to be reached three times, additional names were drawn. All individual face-to-face interviews were conducted in English, audiotaped, and lasted 60 to 90 min. The study participants selected a preferred location on a date and time mutually convenient for both the participant and the researcher. Two of the researchers (SP and GC) conducted the interviews. Information was gathered until saturation was reached [25].

The recruitment of PHNs for interviews began with an open invitation at the three Postpartum Community Services Public Health sites. If they expressed interest, the PHNs completed a demographic form to facilitate purposive sampling, before SR and AD interviewed them. We interviewed a cross-section of 10 PHNs based on their geographic employment location, position based on clinical and/or administrative responsibility (e.g. charge nurse), employment status (full-time, part-time, and casual) and years of experience with Postpartum Community Services. All individual face-to-face interviews were conducted in English, audiotaped, and lasted 60 to 90 min. The study participants selected a preferred location on a date and time mutually convenient for both the participant and the researcher. Information was gathered until saturation was reached [25].

Two researchers independently reviewed verbatim transcripts to identify significant statements, sentences, or quotes and categorized them into clusters of meaning [26, 27]. We used an interpretive thematic analytic approach. Our analysis involved inductive reasoning whereby data gathered from our open-ended research questions were discussed and debated amongst the team that enabled us to pull together data from various parts to compose different themes and represent a whole [28]. We also triangulated data between the mothers and public health nurses. This enabled us to augment the mothers’ experiences and challenges related to breastfeeding. We also used a deductive reasoning approach that permitted de-contextualization and re-contextualizing the data by discussing our understanding of the literature and then moving to our analysis of data [29]. Upon completion of our thematic analysis, the researchers drew conclusions to answer the research questions [30].

### Statistical analysis

We used the Statistical Package for the Social Sciences (SPSS) V23. Feeding was categorized into exclusive breastfeeding, partial breastfeeding, or exclusive formula feeding. Exclusive breastfeeding included mothers who provided only human milk with supplements restricted to vitamins, minerals, or medicine (i.e., no other liquids or solids); partial breastfeeding included those who provided some breast milk regardless of volume [31]. Frequencies and percentages were reported for categorical data. One mother formula fed exclusively, which required her exclusion from the analysis.

## Results

Of the 164 mothers of LPIs eligible to participate, 123 mothers chose to participate. One mother withdrew from the study due to time constraints, leaving 122 mothers in the study. Eighty-four mothers returned their packages, giving us a response rate of 69%. With respect to breastfeeding, we obtained complete data sets for 74 of the 122 mothers.

### Demographic characteristics of mothers and infants

Mothers enrolled in the study had a mean age of 33 years (range 20–47 years). Most of the mothers enrolled in the study were married (*n* = 64, 89%) and graduated from college, trade school, or university (*n* = 44, 59%). Other key demographic characteristics of the parents are presented in Table 1. The self-reported ethnicity of the mothers is presented in Table 1. Demographic information for infants is also presented in Table 1. Twin pregnancies were represented (*n* = 6, 8%) with majority of twins being 36 weeks’ gestational age (*n* = 4, 5%), followed by 34 weeks’ gestational age (*n* = 2, 2%). The major issues at birth were jaundice (*n* = 43, 56%) and feeding difficulties (*n* = 27, 35%).

**Table 1:** Demographic characteristics of parents (*n* = 74) in terms of income, delivery, parity, birth hospital ethnicity and infant characteristics in terms of sex, gestational age, and birth weight (*n* = 80)

	Category	Frequency (*n*)	Percent (%)
Maternal characteristic			
Combined total household income	> $100,000	41	55
	$90,000 to $99,999	7	9
	$80,000 to $89,999	6	8
	$70,000 to $79,999	3	4
	$60,000 to $69,999	8	11
	< $60,000	9	12
Mode of Delivery	Vaginal	51	69
	Cesarean section	23	31
Birth Hospitals	Foothills	34	46
	Rockyview	24	32
	Peter Lougheed	15	20
	South Health Campus	1	1
Parity	Primiparous only	33	45
Ethnic Group of Mothers	Caucasian	48	65
	Mixed/other/missing^a^	8	11
	Chinese	6	8
	Filipino	3	4
	Arab	3	4
	Black/African North American	2	3
	First Nations (registered)	2	3
	Métis	2	3
Infant characteristic			
Sex	Female only	44	55
Gestational age at birth (weeks)	34	20	25
	35	13	16
	36	47	59
Birth weight(grams)	1700–1999	13	16
	2000–2499	23	29
	2500–2999	28	35
	3000–3499	14	18
	3500–3700	2	3

^a^Other ethnicities included South Asian, Southeast Asian, Latino, and West Asian

### Quantitative breastfeeding results

At the time of the study (6–8 weeks after birth) the majority (*n* = 61; 82%) of mothers breastfed their infants. Fifty-one of these 61 mothers partially breastfed their infants and 10 exclusively breastfed. The difficulties experienced by all the breastfeeding mothers appear in Table 2. Of the 13 mothers not breastfeeding at the time of the study, six of them (46%) had breastfed their infant for at least 4 weeks, and five of them (38%) had breastfed between 4 days and 27 days. The reasons cited for discontinuation of breastfeeding included insufficient milk supply (*n* = 6), medical advice received (*n* = 3), discomfort and difficulty (*n* = 2), and antibiotic use (*n* = 1).

**Table 2:** Type of difficulties experienced while breastfeeding

Difficulty	Frequency (*n* = 74)^a^	Percentage (%)
Latch difficulties	30	41
Sleepy infant	36	49
Swollen/painful nipples and breasts	30	41
Not enough milk or flat or inverted nipples	30	41
Maternal Fatigue	31	42

^a^Women could select more than one difficulty

### What is the mother’s experience of caring for LPIs with respect to breastfeeding?

To answer our first research question, researchers interviewed 11 mothers and none appeared distressed or disclosed information necessitating referral for mental health services. Overall, mothers described the desire to breastfeed their babies because “*she was so little, I wanted to make sure she, she got the best nutrients I could give her”* (Mother #10) and because “*it’s work [breastfeeding] but it’s worth it . . . it felt so good to be able to provide something, you know”* (Mother #11). Three major themes were found in the analyses of the mother interviews: significant difficulty with breastfeeding, failing to recognize the infant’s feeding distress and disorganized behavior, and the parental stress caused by the multiple feeding issues.

### Difficulty with breastfeeding: “the biggest issue really has been his feeding”

Feeding represented the most significant issue for mothers. *“The feedings were . . . the most concerning”* (Mother #11). Mother #9 noted that LPIs present special challenges with feeding: *“there is difference. Not like full term baby. Especially in the feeding.”* Mother #8 expressed *“feeling a bit unconfident [with breastfeeding]”* because *“you never know what volume they’re getting.”* Also, the immature development of the infants’ mouth and jaw affected the mothers’ ability to breastfeed. Mother #10 explained, “*she was so little, her mouth was little”* and, thus*, “. . . she just was not latching great.”* Similarly, mother #6 indicated *“we had a hard time with the breastfeeding because she has this really small mouth, and they said that when they’re like early, they don’t suck as well.”* Mother #9 further explained, “*he couldn’t latch well. I think his jaw was still weak. So even after feeding slightly, he stopped; he cannot continue feeding again.”* One mother who had been counseled in the hospital identified feeding problems, such as infants *“forget[ting] how to latch and stuff like that”* (Mother #2). Another described how an infant can *“have her eyes closed and be sleepy in the feed”* (Mother #3).

Even when they wanted their infants to receive breastmilk, they felt conflicted because *“the nurses in the NICU, um, recommend that I breast feed less and bottle more to make sure that he was getting volumes”* (Mother #8). Mother #9 described her concern, *“it [breastfeeding] was really difficult and then after 2 days, he was losing weight more than expected”*. Mothers felt conflicted by the nurses’ recommendations and their personal choices *“They [infants] don’t have enough energy to suck and so the nurse drew the bottle to feed them . . . And I didn’t use a bottle to feed them. I was only breastfeeding them.”* (Mother #7). While mothers were able to recognize specific breastfeeding issues experienced by LPIs, there is room for PHNs to offer more educational support in terms of explicating rationale for specific interventions that were implemented at various points in points.

### Disorganized feeding behavior can lead to feeding distress: “. . . she’s been doing some choking . . .”

Some mothers identified that their LPI demonstrated unanticipated feeding behavior, disorganized feeding behavior, and this sometimes leads to feeding distress. Some LPIs *“got really, really tired out”* (Mother #11). Mother #1 even *“had to physically wake her [infant] which was something [she] wasn’t expecting at all.”* Mother #3 had similar concerns: *“she kind of has been sleeping through feeds”* and is *“sleepy in the feed”*. Mothers noted irregularities in feeding behaviors: *“She kind of would latch on for a bit and then not really . . . it’s probably a bit hard for her to get, you know, her mouth open that wide”* (Mother #11). Mother #3 explained that she discussed her feeding concerns with her physician:

The other feeding issue that we’ve had is choking . . . more so . . . with the bottle at first and . . . when I moved to a larger nipple shield and it started happening on the breast. I talked to my doctor about it and really she said it’s like, my flow . . . Like I’ve produced a lot of milk and so it’s just kind of a little bit too much for her.

Other feeding issues included trouble establishing an effective latch, poor coordination of suck and swallow, and disorganized feeding behavior. Mother #5 explained:

So, there was like this sucking issue, and obviously he wasn’t latching really well . . . it just felt like he was really disorganized. Like he couldn’t quite coordinate himself to do it . . . he would have couple feeds where he would do well . . . and then he’d have a couple where . . . he was tired or he just, you know, didn’t seem to be able to do it and then he would have more of the supplement.

All mothers shared feeding experiences that appeared unfavorable either for the LPIs, mothers or both. Mothers did not speak to what interventions they attempted to assist in remedying the feeding issues. Therefore, it is important for health care providers to explore unfavorable feeding situations and offer the appropriate teaching with helpful interventions that support breastfeeding.

### Feeding issues caused parental stress: “it was very frustrating”

Due to the frequency and duration of these challenges, feedings became prolonged, and mothers became exhausted. Parents could not understand why *“our very relatively healthy baby was having feeding issues”* (Mother #5). Mothers found that managing the feeding issues became *“a lot of work,”* (Mother #7) and was *“exhaust[ing]”* (Mother #9). Mother #1 explained how and why *“It was very frustrating”*:

She would not latch because she was preterm and it took a lot of work . . . so I saw a lot of different people to try to get her to latch, but she wouldn’t so it was very stressful and frustrating for me and I can just imagine that it was probably just as frustrating for her . . . All that matters is that she gets breast milk . . . [how] she gets it isn’t a big deal to me . . . But no one seemed to care how I felt or what I thought. They just thought breastfeeding was the best, and I didn’t care how she got it, if it was in a bottle or whatever (laughing), but they seemed to think that the bottle wasn’t the way to go.

Some encounters with health care providers also contributed to parental anxiety. Mother #3 did not feel supported in her choice to breastfeed:

Um, my milk didn’t come in obviously for a couple days, and they really pushed getting her enough to . . . to keep the jaundice away, which, I think, overall was like, a good decision, but also I kind of felt like my choices were sort of being taken away from me, and it was more like pushed onto us that we should have her have formula. And I really didn’t want her to.

While health care providers implement interventions to promote the overall health of LPIs, there are potential areas where health care providers can explain rationale for specific interventions so that parents feel supported in caring for their LPI.

The contradictory advice given to Mother #5 “upset” her:

I did cry. I feel like every person I’m seeing I’m getting different advice from. And I’m finding that really frustrating. I feel like honestly every time I come in, I’m being told what I’m doing wrong and what I’m doing is what I’m being told to do.

Mother #3 indicated that a lot of distress resulted from a lack of anticipatory guidance about the challenges of feeding LPIs.

I guess more explanation for us about the feeding. Like, everybody kind of sent us home on a plan, but nobody really explained to us that this is normal and that there can be these sorts of issues, and, um, what to expect. We didn’t really hear that . . .

The challenges that mothers of LPIs experience are varied and include latching difficulty, feeding distress, disorganized feeding behaviour, and interactions with diverse health care providers wherein conflicting advice was received. Furthermore, some mothers perceived a lack of support or no information about what to expect when caring for an LPI. All these experiences pose a considerable amount of stress for mothers.

### What is the PHN’s experience of caring for LPIs with respect to breastfeeding?

To answer our second research question, researchers interviewed 10 PHNs. In their interviews, the PHNs spoke at length about the challenges of guiding mothers in feeding LPIs. Our analysis revealed three themes: challenges with initiating breastfeeding, challenges while breastfeeding, and increased need for skin to skin contact and other forms of stimulation while feeding.

### The complexity of initiating a successful breastfeeding routine: “initiation [of breastfeeding] is crucial”

PHNs commented on the complexity of initiating a successful breastfeeding routine. In her experience, PHN #3 observed: *“That initiation [of breastfeeding] for all women is crucial. It’s crucial. It’s their own confidence and their own ability to latch and recognize when their baby is feeding well . . .”*. The challenges of initiating breastfeeding often stem from the health care culture (PHN #5). PHN 5 believed *“these hospitals are not baby friendly”*. She explained:

And, I find it very different in this setting in this city where formula is readily accessible and available and they stick it there [in front of the mothers]. And so then I find a lot of times people are just saying. ‘oh well, you know, I’m not going to be able to breastfeed this baby.’ They’ve been given the formula in the hospital and so they just carry on mixed feeding. So breastfeeding in itself is not as encouraged . . . And there’s also misconception around what’s easier, bottle or breast. And a lot of times babies at this age and gestation are being given bottles and [mothers are] being told that they should have bottles because it’s easier for the baby. Yes and no . . . Because bottles in themselves can actually cause trouble for these babies . . . When babies are not on the right flow . . . they can be choking, and sputtering . . . and then that bottle experience is now a difficult bottle experience leading to more troubles . . . whereas breastfeeding, it’s comfortable. They can control the flow, yeah. And even though they can get tired, you can . . . finish the breastfeeding by doing it more frequently or you can, you know, maybe supplement with the bottle at the end.

While the initiation of breastfeeding is critical, it is clear that the process for initiating breastfeeding for mothers of LPI requires a coordinated approach by all health care professionals who encounter mothers and their LPIs in the immediate postpartum period. Breastfeeding LPIs requires a unique type of support from PHNs after discharge from hospital.

### Breastfeeding challenges: “every feeding is different”

In part, the complexity of breastfeeding derives from the lack of any specific method to measure consumption of breast milk. Some mothers, based on instructions they receive, limit the frequency and duration of the breastfeeding to preserve their infants’ energy. PHN #5 explained:

People are looking at the time limit. So the moms got the baby at the breast saying 20 min of feeding, and the baby might not be doing anything . . . The baby might not even be sucking . . . So the mom puts the baby to the breast for 20 min then gives it a bottle. The baby takes 60 mL by bottle and goes to sleep and the baby is not actually breastfeeding.

Working with mothers to understand the reasoning behind inconsistent feeding patterns and the associated cues was also a challenge. PHN #4 acknowledged that *“. . . every feeding is different . . . So your baby might be really alert and actively sucking and swallowing at this feeding, but the next one, he’s not doing anything.*” Furthermore, “*they have to eat more frequently, and you need to encourage the parents about that need . . . I actually don’t know how to really impart that to a lot of parents”* (PHN #1). In addition, *“[parents] don’t realize that sometimes [LPIs] tire very easily . . . and the parents often think that ‘Oh, they’re done, they’re full’ and in fact they’re not. They’re just kind of exhausted.”* (PHN #2). PHN #3 expanded on this theme when she stated, *“So they can suck and suck and suck and then it is like they are running a marathon . . . And they could become distressed, and it can predispose them to um becoming stressed with feeding.”* PHN #4 added that it is important for mothers to recognize infants’ cues *“. . . like when they’re shutting down”*.

Helping mothers to achieve the goal of exclusive breastfeeding presented another challenge. PHN #3 explained, “we need them to know this takes time and were going to help them . . .We are teaching about hunger and cessation cues and . . . we want to give them confidence”. PHN #4 explained exclusive breastfeeding “absolutely could happen; it just really depends on how motivated the mom is.”

### Supporting the LPI during breastfeeding: “kind stimulation, gentle stimulation”

LPIs sometimes require skin-to-skin contact and other forms of stimulation when breastfeeding. PHN #3 found that *“different hospitals do different things . . . lots of skin-to-skin, and trying to get the baby to the breast within an hour. Um that that really makes a difference.”* PHN #4 indicated that *“learning how to breastfeed, you really need to do lots of skin to skin so I really promote that and encourage them to . . . continue doing that as often as they can.”* While early skin-to-skin contact is preferred, PHN #3 noted that the hospital setting can interfere when nurses appropriately encourage mother-infant interactions immediately after birth:

These babies perhaps need more resuscitation. They may need more pediatric input at delivery. They might be separated from mother for longer at delivery. Worst case scenario they are in ICU . . . We know that’s an enormous barrier to early bonding, early breastfeeding. And that’s probably a big part of the piece with these babies, because they are far more likely to be poked and prodded and resuscitated . . . The unpleasant painful stimuli for these babies – we know that these babies that have had tubes and whatever down their throat, down their nose . . . deep suction . . . does nothing to assist early breastfeeding.

In their interviews, PHNs emphasized the importance of *“kind stimulation, gentle stimulation”* (PHN #1). *“We always do stimulation, and tickling toes, rubbing palms, stroking your baby in some way, you know to wake them up . . .”* (PHN #3). *“Warming up the little blanket seems to help those little babies quite a bit too. You can’t put a big mouth crying, pushing, onto a mother’s breast – that’s not going to work. The baby needs to be calmer.”* (PHN #1). Additionally, she stressed the importance of appropriate stimulation. For example, it’s not suitable to *“put a cold washcloth on these little babies anymore thank goodness”*, and *“we must make sure they don’t [blow dry the babies’ feet] because of the possibility of burns . . . but there are people that do that”*. PHN #3 complained of the practice to encourage crying *“. . . in order to make them feed more is really um quite mean actually. It’s quite mean.”*

## Discussion

Most of the mothers in our study partially breastfed their infants at 6–8 weeks postpartum (82%) and a very small proportion (14%) exclusively breastfed at 6–8 weeks after the birth. Nagulesapillai and colleagues found that 55% of mothers of LPIs exclusively breastfed at 4 months in Calgary [12]. The discrepancies may be attributed to the fact that the difficulties encountered during the early breastfeeding experience may have been overcome by the time the LPIs were 4 months of age. Nevertheless, many studies have demonstrated that a late preterm birth is foretelling of breastfeeding difficulties or breastfeeding failure. LPIs seem at greater risk of not being able to breastfeed successfully or breastfeed exclusively at hospital discharge, compared with infants born at 37 weeks gestation [21, 32, 33].

The qualitative results of our study indicate that mothers find breastfeeding challenging and PHNs find it difficult to guide mothers in breastfeeding. Generally, mothers in our study attribute the challenges to their infants’ prematurity. Notwithstanding the validity of these attributions, mothers and PHNs should also be able to consider the complexity of sucking, swallowing, and breathing, often done simultaneously, during feeding [34]. LPIs have to coordinate all of these psychomotor activities, and the ability to do this effectively is related to the gestational ages of the infants [35]. The PHNs have a role that requires more study and thought.

On a related matter, parents of LPIs and PHNs should understand the relationship between brain development and feeding. At 34 weeks gestation, the LPI brain weighs only 65% of the brain of a term infant [36]. More than one third of brain growth occurs in the last 6–8 weeks of gestation and it is during this time period that the white matter increases 5-fold [36, 37]. Equally important, during the last 10 weeks of gestation, the brain undergoes a 4-fold increase in grey matter [36]. Consequently, during the final weeks of gestation, the oral motor skills become more coordinated and periods of alertness become more predictable [38]. Undoubtedly, significant brain development occurs during the last few weeks of gestation. This fact suggests that neurodevelopmental maturation, in addition to experience or learned behavior, contributes significantly to the feeding behaviors of LPIs [39]. For example, LPIs who have difficulty latching on to the breast correctly could likely have immature sucking and swallowing reflexes [34]. Parents and PHNs should understand the unique feeding challenges due to the relationship between gestational age and brain development. Whether this neuro-cognitive and psychomotor development also explains any greater success of mothers in breastfeeding their infants exclusively requires further study. However, the fact that mothers can rightfully expect such development to occur provides PHNs with facts that should give mothers hope for improvement.

Mothers and PHNs recognize that LPIs sometimes exhibit a lack of alertness, which interferes with breastfeeding. Alertness profoundly affects any interest in feeding as well as feeding performance [38]. A healthy term infant has a well-developed sleep-wake cycle that allows for periods of alertness and deep sleep within the first 36 h of birth [38, 40]. This sleep-wake cycle allows healthy term infants to wake when hungry, remain alert during feedings, and then transition back into a deep sleep until the next feeding [38]. In comparison, LPIs have more difficulty achieving a deep sleep, and therefore lack sufficient rest to remain alert for the subsequent feeding [38]. Consequently, LPIs could appear unable to maintain a state of arousal and expend the required energy for adequate feeding [41]. When compounded by other factors (temperature instability, respiratory distress, apnea, hypoglycemia, jaundice, and sepsis) LPIs also have a decreased state of arousal and poor endurance that could lead to early fatigue during feeding [34]. The PHNs can assist parents in understanding the physiological mechanics of fatigue.

Fatigue may be revealed through observing muscle tone during feeding. LPIs exhibit fairly mature muscle tone on initial examination, but parents and caregivers should notice that muscle tone can diminish, much sooner than in a term infant, as demonstrated by arms falling limp quickly during a feeding, and this would indicate decreased stamina [38]. Oftentimes, caregivers interpret fatigue as satiation [17]. Such thinking can have untoward consequences, if they make the wrong inference and preemptively end the feeding. It is imperative for mothers not only to recognize and understand states of arousal and the effect of fatigue, but to be able to recognize cues from the LPI that demonstrate arousal and fatigue, and the subsequent impact that arousal and fatigue have on breastfeeding success.

Whereas the length of the postnatal hospital stay has decreased over the last few decades, questions about the safety of this practice persist [42]. The focus on early discharge could interfere with breastfeeding success for the LPI population, with the infant taking enough by mouth during the postnatal hospitalization period only to have feeding volumes decrease after discharge. Not unlike the breastfeeding mothers of term infants, the breastfeeding mothers of LPIs report difficulty in discerning whether their infants received sufficient milk. Whether this occurs as the result of any lack of anticipatory guidance and/or reduced breastfeeding support on the part of providers or community support remains open to question [43]. Whatever the reason, the problem of ascertaining insufficient feeding persists [38]. Such was the case with the mothers we interviewed.

Mothers and PHNs in our study acknowledged that mothers received conflicting information from various health care providers. This likely occurs due to the lack of formal training that health care providers receive about breastfeeding. Pound and colleagues identified several areas of knowledge deficits with respect to breastfeeding among Canadian physicians [44]. Not unlike their medical colleagues, PHNs and other health care professionals could also benefit from additional training [16, 43–45]. They should receive training in identifying infant cues associated with feeding distress and then implementing successful remedies. In the same spirit of sharing knowledge, providers should also learn strategies on how to provide anticipatory guidance on the subject. With this new knowledge, health care providers can educate mothers and other caregivers about infant cues that suggest breastfeeding success. Of course, this recommendation presupposes that the matter receive more research.

A lack of coordination and care represents another reason why mothers and families receive conflicting advice from health care providers. Kurth and colleagues stress the importance of health professionals being better connected to one another; to work together collaboratively to provide the type of care that families require after early discharge [46]. Given the myriad of breastfeeding challenges experienced by mothers and the challenges experienced by PHNs who guide mothers in breastfeeding, improved coordination of care could also prove useful when working with LPIs in the community setting. Providing a seamless transition between services provided in the hospital and services provided in the community is important to coordinate strategies that will improve breastfeeding outcomes [43, 47]. Kurth and colleagues offer building a continuum of care from pregnancy to the postpartum period wherein various health care providers would be linked in a systematic manner [46]. Such an approach would leverage existing organizational units and adapt communication processes to transfer relevant information between acute care and community based health services in a timely fashion [46]. It is therefore clear that mother-infant dyads would benefit from greater education and support, and improved coordination of care to optimize breastfeeding outcomes for infants and improved breastfeeding experiences for mothers. The findings of our study should be interpreted with caution given the limitations of our sample. Firstly, we used a convenience sample, therefore it is unclear how representative our quantitative descriptive data is of the general population of mother with LPIs. Secondly, our results likely do not reflect the experiences of less educated mothers or minority women. Finally, this study was limited to women who could read, write, and speak English fluently and therefore may impact the generalizability of our study findings.

## Conclusions

The breastfeeding journey for mothers of LPIs can follow a complicated course. We found that mothers of LPIs experienced significant difficulty with breastfeeding. The multiple feeding issues experiences caused much stress for the mothers. PHNs articulated the complexity of initiating a successful breastfeeding routine with LPIs. PHNs also noticed that mothers had challenges recognizing hunger and cessation cues during breastfeeding. The support that is required for LPIs in terms of stimulation during breastfeeding was highlighted. From our research, we identify that much more can be done to support mothers in breastfeeding their LPI. It is necessary that all health care professionals working with LPIs and their families receive the appropriate level of training with regards to safe and effective breastfeeding. It is equally important for parents and families of LPIs to be aware of the breastfeeding challenges that they may potentially experience. A seamless transition from the acute care to the community setting, with appropriate coordination of care, is imperative to promote healthy growth and development and to prevent feeding-related morbidity and mortality associated with LPIs.

## Abbreviations

LPI: Late preterm infants
PHN: Public health nurse
